# Origins of Moiré Patterns in CVD-grown MoS_2_ Bilayer Structures at the Atomic Scales

**DOI:** 10.1038/s41598-018-27582-z

**Published:** 2018-06-21

**Authors:** Jin Wang, Raju Namburu, Madan Dubey, Avinash M. Dongare

**Affiliations:** 1 Department of Materials Science and Engineering and Institute of Materials Science, University of Connecticut, Storrs, Connecticut 06269 USA; 2 Computational and Information Sciences Directorate, U.S. Army Research Laboratory, Aberdeen Proving Ground, Aberdeen, Maryland 21005 USA; 3 Sensors and Electron Devices Directorate, U.S. Army Research Laboratory, Adelphi, Maryland 20783 USA

## Abstract

The chemical vapor deposition (CVD)-grown two-dimensional molybdenum disulfide (MoS_2_) structures comprise of flakes of few layers with different dimensions. The top layers are relatively smaller in size than the bottom layers, resulting in the formation of edges/steps across adjacent layers. The strain response of such few-layer terraced structures is therefore likely to be different from exfoliated few-layered structures with similar dimensions without any terraces. In this study, the strain response of CVD-grown few-layered MoS_2_ terraced structures is investigated at the atomic scales using classic molecular dynamics (MD) simulations. MD simulations suggest that the strain relaxation of CVD-grown triangular terraced structures is observed in the vertical displacement of the atoms across the layers that results in the formation of Moiré patterns. The Moiré islands are observed to nucleate at the corners or edges of the few-layered structure and propagate inwards under both tensile and compressive strains. The nucleation of these islands is observed to happen at tensile strains of ~ 2% and at compressive strains of ~2.5%. The vertical displacements of the atoms and the dimensions of the Moiré islands predicted using the MD simulation are in excellent agreement with that observed experimentally.

## Introduction

Two-dimensional (2D) transition metal dichalcogenide (TMD) structures, with their unique electronic structure, show significant promise for applications in field effect transistors^1^, optoelectronic device^2^, photo transistors and photo detectors^3^. Bulk MoS_2_ is characterized by an indirect band gap with an energy of 1.29 eV using absorption and photoluminescence (PL) spectroscopy measurements. This band structure corresponds to a valence band maximum (VBM) at Γ and the conduction band minimum (CBM) at Σ_*min*_^4^. When bulk MoS_2_ is thinned to monolayer, the VBM shifts from Γ to K, and the CBM shifts from Σ_*min*_ to K, which results in an intriguing indirect-to-direct transition of band gap energies for monolayer MoS_2_. While only the monolayer MoS_2_ exhibits a direct band gap of 1.9 eV, few-layered MoS_2_ structures in addition to monolayer MoS_2_ also render a faster electron-hole recombination process attributed to quantum confinement effects as compared to bulk MoS_2_ crystals^5^.

Moreover, the electronic properties of 2D MoS_2_ structure are remarkably reconstructed under the external strain^6–11^, which opens up the opportunity to tailor the performance of these materials for device application^12–15^. As a result, several experimental studies have investigated the strain response of 2D MoS_2_ structures. For example, PL and absorption spectroscopies measurements reveal that the band gap (E_g_) of a monolayer or bilayer MoS_2_ sample decreases significantly upon a moderate in-plane uniaxial extension that is applied by bending the substrate^6,7,9^. Similar reductions in band gap energies have been reported for trilayer MoS_2_^16^, as well as under strain conditions normal to the layers^17^. A significant number of theoretical studies have also been carried out to understand the variations in the electronic band structures of 2D MoS_2_ structures^18–23^. These studies suggest a transition from a direct band gap to an indirect band gap for monolayer MoS_2_ at ~ 2% tensile strains and a semiconductor to metallic transition at ~10% biaxial strain^18^. Several transitions for the indirect band gap are also observed for various strains for the bilayer MoS_2_ systems^19^. These band gap transitions are observed to be consistent with the PL and absorption spectrum measurements that demonstrate strong blueshifts in E_g_ of 2D MoS_2_ under the presence of uniaxial strain^6,7^.

An interesting aspect of the strain response of few-layered 2D structures is that the weak van de Waals (vdW) interactions between substrate and 2D structures can render variations in the strain response of the 2D structures. For example, the strains in top layers of the multilayered MoS_2_ structures may be relaxed through nucleating ripples^24–26^ or sliding between the layers^27^. These ripples are attributed to the presence of edges in chemical vapor deposition (CVD)-grown MoS_2_ structures that result in differences in the strains across the layers^24^. In addition, the vertically heterogenous strain configurations across layers can induce/engender lattice mismatch between different layers, which results in a Moiré-like superstructure in individual layers, i.e., Moiré patterns^28^. The lattice-mismatched Moiré-pattern structures are considered as the evidence of successful alignment/growth of heterostructures, and have been extensively observed in many vdW heterostructures^29–33^, graphene-metal interface^34^ as well as MoS_2_-metal interfaces^35–38^. For example, the graphene-hBN heterostructure with a lattice mismatch of 1.8% shows a Moiré pattern with a well-defined periodicity of ~15 nm^29–31^, which is confirmed by continuum models^39^. These Moiré superlattices are reported to have a non-uniform out-of-plane displacement in the magnitude of ~0.2 nm^39^. A relatively large Moiré distance of ~ 30 nm is reported for large-area single layer MoS_2_ (SLMoS_2_) grown epitaxially on Au (111) surface^35-38,40^. Due to the in-plane nature of the orbitals in MoS_2_, the interaction between metal substrate and SLMoS_2_ has less pronounced consequences on the electronic structure as compared to graphene-metal interface^40^. However, the band structure of SLMoS_2_ near the K-point are evidently affected and a noticeable Mo 4d state is observed^40,41^.

The recent progress in CVD growth method is able to prepare 2D MoS_2_ structures with large area and high quality, and therefore is a breakthrough approach in fabrication of micro-scale 2D MoS_2_ for the application devices^42–44^. These CVD-grown structures comprise of multiple layers with unequal dimensions. For example, the CVD-grown bilayer flake^45^ comprises of a bottom layer as a triangle and the top layer that is also a triangle with smaller dimensions and 180^0^ rotation angle as compared to the bottom layer. The strain response of these structures suggests that the few layer MoS_2_ structures is characterized by shifts in both the Raman in-plane mode and photoluminescence (PL) energy^27^. On the other hand, Moiré patterns have been observed in 2D materials grown by CVD method. For example, the Moiré pattern with a periodicity of around 14 nm has been identified when monolayer or bilayer graphene were grown on *h*-BN substrate using CVD method^31^. Also, the Moiré pattern observed in CVD-grown MoS_2_/WSe_2_ hetero-bilayers indicates a periodicity of 8.7 nm^33^. The Moiré-like superstructures exhibit distinct electronic properties as compared to the constituent layers. For example, it has been observed that the Moiré structure in MoS_2_-WSe_2_ bilayer presents a periodic local-bandgap variation with an amplitude of ~ 150 meV and the band edges are observed to be located at different layers^33^. Thus, the formation of Moiré patterns can play a vital role in altering the electronic properties and mechanical response (such as strain relaxation) of CVD-grown MoS_2_ structures.

**Figure 1:**
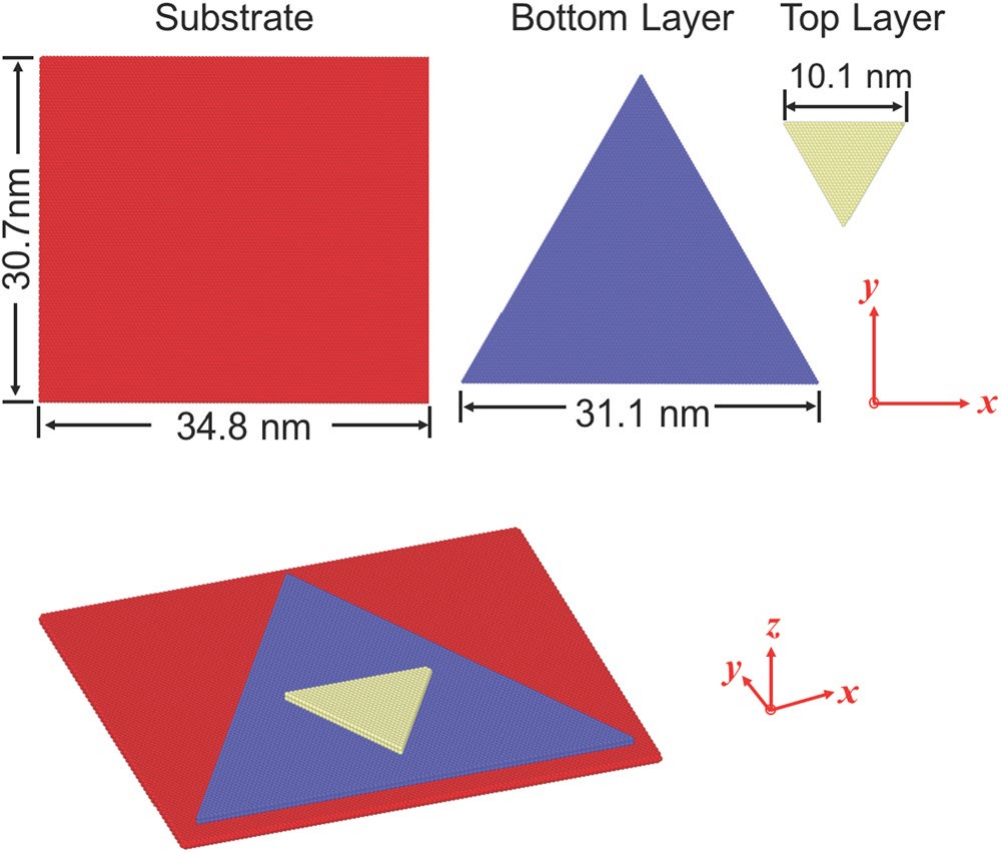
The structure of triangular MoS_2_ bilayer with a substrate. Top row: the top view of substrate, bottom layer and top layer; middle row: side view; bottom row: front view.

This study investigates the mechanisms of formation of Moiré patterns under applied strains in CVD-grown bilayer MoS_2_ structures using molecular dynamics (MD) simulations (see method section and supplemental information). The CVD-grown MoS_2_ triangular flakes comprise of few layers in dimensions of a few microns wherein the top layers are smaller triangles with a rotation angle of 180^0^ as compared to the bottom ones. Strain is applied to a triangular bilayer system using a substrate comprising of a large rectangular MoS_2_ layer to mimic a CVD-grown flake supported on a substrate. The bilayer triangular system with a rectangular substrate is presented in Fig. 1. 2H stacking sequence is used in all the three layers. The substrate layer is a rectangle in the basal plane with the dimension of 34.8 nm × 30.7 nm. The bottom layer and top layer are both equilateral triangles with a side length of 31.1 nm and 10.3 nm, respectively. The center of top layer is right above that of the bottom, and the top layer rotates 180^0^ with respect to the bottom layer. Periodic boundary conditions are adopted for the system. A vacuum space of 10 nm thick is used along the vertical direction of the system to prevent unphysical interactions between adjacent images along this direction. This model provides realistic edge configuration as observed in the CVD-grown MoS_2_, and allows the investigation of the strain response of the triangular shaped flakes with substantial amount of edge.

It is assumed that the strain is directly subjected to the substrate layer. As a result, the forces on the atoms in the substrate are rescaled to zero at each time step, so that the atoms are prohibited to move and the strain in the substrate atoms is exactly identical to the applied strain. The strain response is investigated under the loading condition of biaxial strain (*ε*_*x*_ ≠ 0, *ε*_*y*_ ≠ 0, *σ*_*x*_ ≠ 0, and *σ*_*y*_ ≠ 0). Both tensile and compressive strain are studied. The biaxial strain is applied along the X and Y direction in increments of 0.1% and the system is allowed to relax to minimize the total energy of the system at each increment. The straining is continued to reach a total strain of 6% which is within the elastic region for the MoS_2_ system using the REBO potential^46^. At each increment, a layer strain along X and Y direction ($\,{\varepsilon }_{x}^{{L}_{i}}$ and ${\varepsilon }_{y}^{{L}_{i}}$) is calculated for each individual layer as

1 $${\varepsilon }_{x}^{{L}_{i}}=\frac{{x}_{i}-{x}_{i0}}{{x}_{i0}},\,{\varepsilon }_{y}^{{L}_{i}}=\frac{{y}_{i}-{y}_{i0}}{{y}_{i0}}$$

where, *i* is the layer-id (1, 2; 1 corresponds to bottom layer and 2 is top layer); ${x}_{i}$ (${y}_{i}$) and ${x}_{i0}$ (${y}_{i0}$) are final and initial layer length of *i*^th^ layer in X (Y) direction, respectively. The analysis of the layer strain provides the strains of each individual layer and enables the investigation of the relaxation of each layer in each direction during the straining process.

## Results and Discussions

**Figure 2:**
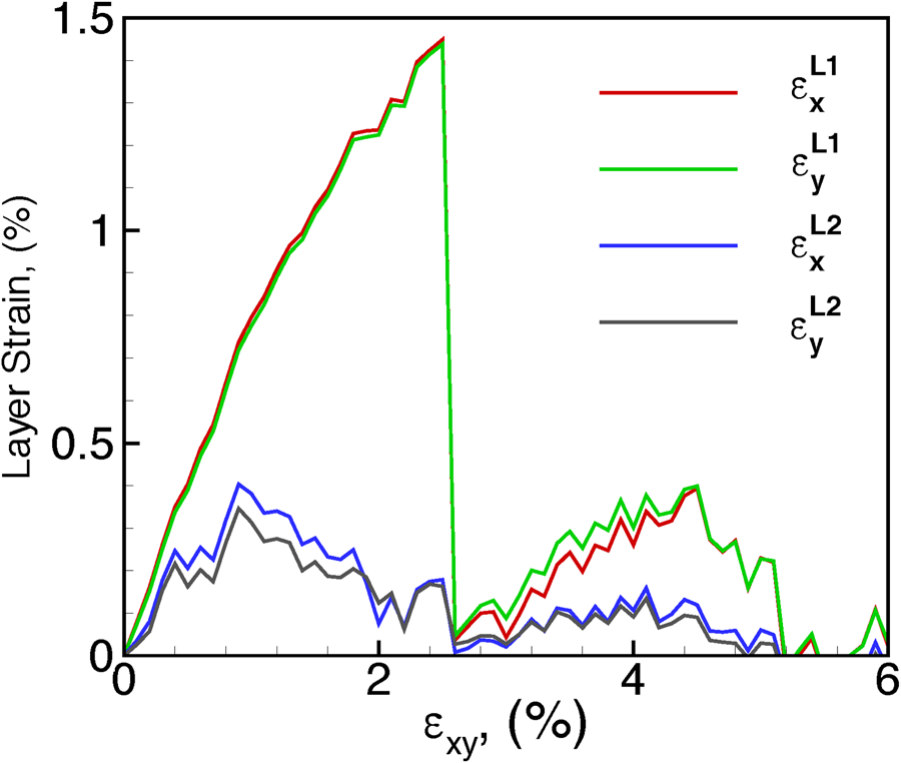
Local layer strain ${\varepsilon }_{x}^{top}$, ${\varepsilon }_{y}^{top}$, ${\varepsilon }_{x}^{bottom}$, ${\varepsilon }_{y}^{bottom}$ as a function of applied tensile strain $\varepsilon$ show the emergence and nucleation of Moiré island under biaxial tensile strain.

The variations of ${\varepsilon }_{x}^{{L}_{i}}$ and ${\varepsilon }_{y}^{{L}_{i}}$ are plotted as a function of the applied biaxial tensile strain (referred to as *ε*_*xy*_ to indicate *ε*_*x*_ = *ε*_*y*_) for each individual layer, i.e., bottom layer and top layer, in Fig. 2. The layer strain in X and Y direction are almost the same for both bottom and top layer, which indicates the strain relaxation is direction-independent. It can be seen that the layer strain in the bottom layer and the top layer both present a periodical fluctuation every ~2% applied strain. At a small value of applied strain, both the top layer and bottom layer show a linear increase with *ε*_*xy*_, but, it should be pointed out that the strain subjected to the substrate is not completely transferred to the bilayered MoS_2_ triangles due to the weak van der Waals interactions between the substrate and the MoS_2_ layers. For example, the layer strain in *x* direction of the bottom layer is ${\varepsilon }_{x}^{{L}_{i}}$ = 0.8% at the applied strain of *ε*_*xy*_ = 1%. As a result, the layer strain in the bottom MoS_2_ layer shows about 20% relaxation at this stage. The top layer presents more noticeable relaxation. Such an incomplete strain transfer between substrate and the MoS_2_ bilayers renders a lattice mismatch between the layers. The layer strain continues to increase with applied strain for each layer up to a certain value, after which a further increase in applied strain results in a sharp drop in the local strain. Further straining results in an increase in layer strain again up to a certain value followed by a sharp drop again. However, the peak strains in the 2^nd^ cycle are observed to be significantly lower for each layer as compared to the 1^st^ cycle. The layer strain in the bottom layer is consistently higher than that in the top layer, suggesting a larger relaxation in the top layer due to the presence of a free surface above the top layer. However, it is not clear if these simultaneous variations are related to the sliding between the layers or related to the presence of Moiré patterns.

**Figure 3:**
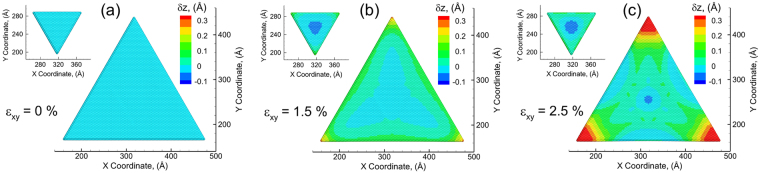
The vertical displacement map in the top layer and bottom layer at different applied biaxial tensile strain: (**a**) 0%, (**b**) 1.5%, and (**c**) 2.5%. The atoms are colored by the displacement in the Z direction with the positive value (red color) corresponding to an upward displacement and a negative value (blue) indicating a downward displacement as compared to the unstrained configuration.

To investigate the strain relaxation mechanisms of the various layer strains, an out-of-plane vertical displacement is computed for each atom along the length of layers in the bilayered structure. This vertical displacement ($\delta {z}_{i}$), at a certain atom *i*, is computed as $\delta {z}_{i}={z}_{i}-{z}_{0i}$, where ${z}_{i}$ and ${z}_{0i}$ are the instantaneous and initial Z-coordinate of atom *i*, respectively^24,25,39,47^. The vertical displacement will be expected to be uniform for the case of sliding between the layers as the strain relaxation mechanism. For the case of the emergence of Moiré pattern, a vertical displacement profile will show high displacements at the edges of the top layer and bottom layer. The map of $\delta {z}_{i}$ are shown in Fig. 3 to visualize the local variations in vertical displacements at different applied strain at the atomic scale. The atoms are colored by the displacement in the Z direction with the positive value (red color) corresponding to an upward displacement and a negative value (blue color) indicating a downward displacement as compared to the unstrained configuration. The strain-free configuration (*ε*_*xy*_ = 0%) shows no displacement along vertical direction ($\delta {z}_{i}$ = 0) for all atoms (colored as light blue) as shown in Fig. 3(a). At an applied strain value of *ε*_*xy*_ = 1.5%, the atoms in the corner of bottom layer triangle are displaced upwards (yellow/orange) and the atoms in the center are gradually displaced downwards (dark blue) as shown in Fig. 3(b). The maximum upward and downward displacements of the bottom layer are computed to be 0.28 Å and −0.03 Å, respectively. While there is no upward displacement in the top layer, the maximum downward displacement of the top layer is −0.053 Å, which is larger than that in the bottom. The corner of the top layer is also displaced upward as much as 0.087 Å. It is intriguing to mention that the downward displacement pattern found at *ε*_*xy*_ = 1.5% is incredibly similar to the PL intensity map of stacked bilayered triangular system under a strain (Fig. 4(c) in Zheng *et al*.^25^), where the PL intensity of bottom layer is low at the center and increases with a sharp tail towards the corner (a shape of three-pointed star), while the PL intensity of top layer is noticeably low at the center as compared to the bottom layer and slightly high at the edge^25^. The comparable results further suggest that the current interatomic potential is capable to capture the strain response of the bilayer system. At the further strain of *ε*_*xy*_ = 2.5% (Fig. 3(c)), the bottom layer shows more upward displacement at the corner with an enhanced magnitude of 0.39 Å, and the tail of the “three-pointed star” turns to be broad. It is noticed that the edges of the bottom layer present a downward displacement around −0.024 Å. Besides, the downward displacement of the center in bottom layer also increases, with a maximum of −0.056 Å.

**Figure 4:**
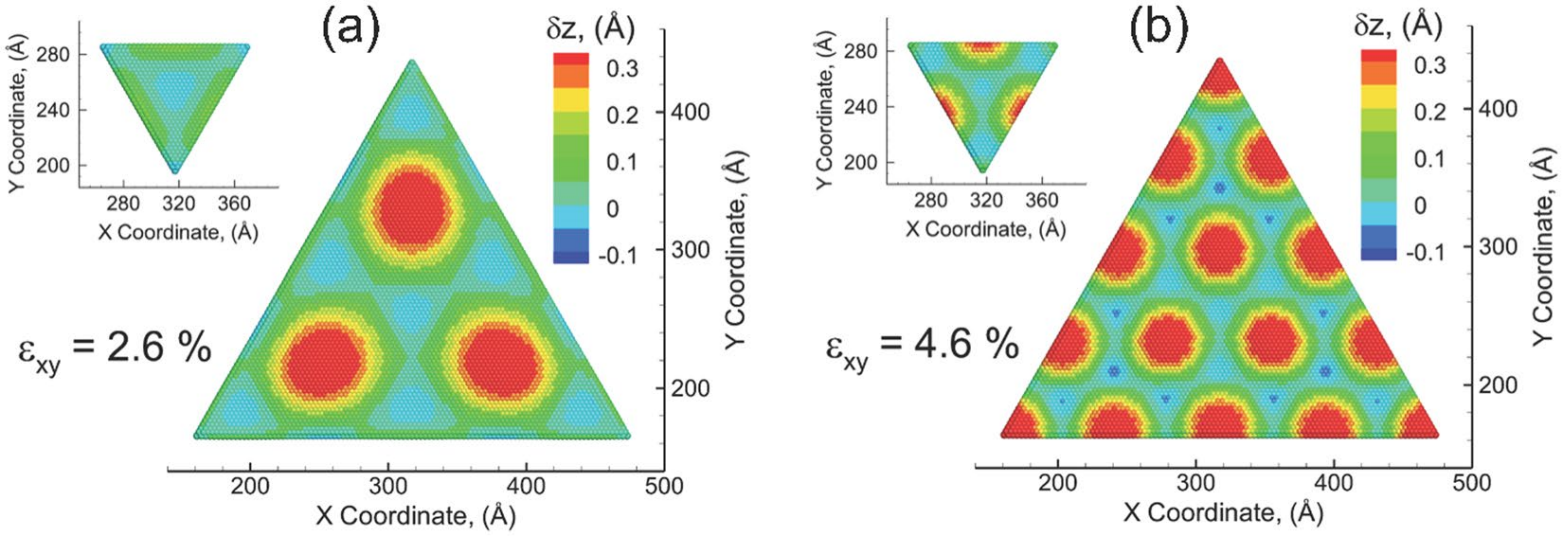
The vertical displacement map shows the nucleation of displacement islands and the formation of Moiré pattern in the top layer and bottom layer at different applied biaxial tensile strain: (**a**) 2.6%, (**b**) 4.6%.

At an applied strain of *ε*_*xy*_ = 2.6% (Fig. 4(a)), where the layer strain undergoes a substantial reduction as shown in Fig. 2, the downward displacements at the corner shift to the center and form three displacement islands (i.e. Moiré patterns). The dramatic decrease in the layer strain is attributed to the emergence of such Moiré-like patterns. As listed in Table 1, the maximal upward displacement in the center of the Moiré island is observed to be 0.42 Å, which is comparable to those observed in MoS_2_-WS_2_ (0.51 Å) or MoS_2_-WSe_2_ (0.39 Å) heterostructures from continuum simulations^39^. The periodicity of the Moiré pattern is 13.09 nm as computed from the distance of two Moiré islands, which is slightly larger than that in MoS_2_-WS_2_ bilayer, ~8.7 nm, and lower than that in MoS_2_-Au (111) interface, ~30 nm^35–38,40^. For a bilayer system with a lattice mismatch δ and misoriented angle φ, the periodicity of the Moiré pattern is given by^31^

2 $${\rm{\Delta }}=\frac{(1-\delta )a}{\sqrt{2(1-\delta )(1-cos\phi )+{\delta }^{2}}}$$

where *a* is the lattice constant of the bottom layer. In this study, the Moiré patterns forms because of the lattice mismatch between substrate layer and the MoS_2_ bilayer. There is no mis-oriented angle between substrate layer and the MoS_2_ bilayer, i.e. φ=0. As a result, the periodicity is

3 $${\rm{\Delta }}=\frac{1-\delta }{|\delta |}{a}_{sub}$$

where δ is the lattice mismatch between substrate layer and MoS_2_ bilayer (we use bottom layer of MoS_2_ bilayer), and *a*_sub_ is the lattice constant of the substrate layer. The lattice mismatch is determined from the lattice constant of the substrate layer and bottom layer of MoS_2_ bilayer, which can be calculated from the unstrained MoS2 lattice constant *a*_o_ = 0.317 nm, *ε*_*xy*_, ${\varepsilon }_{x}^{{L}_{i}}$ and ${\varepsilon }_{y}^{{L}_{i}}$. At an applied tensile strain *ε*_*xy*_ = 2.6%, the lattice mismatch between substrate layer and MoS_2_ bilayer is 0.0249, and *a*_*sub*_ = 0.325 nm. As a result, the theoretical periodicity of Moiré pattern is 12.72 nm which agrees very well with the value of 13.09 nm observed with MD simulations. No Moiré pattern is observed in the top layer at the current applied strain. However, it should be noticed that the edges of the top layer undergo a recognizably large upward displacement, 0.13 Å as indicated by the noticeable green color on the edges in Fig. 4(a). Meanwhile, the downward displacement in the center of the top layer slightly decreases to −0.037 Å as compared to −0.056 Å at *ε*_*xy*_ = 2.5%. As shown in Fig. 4(b), at the further straining of the structure to an applied strain of *ε*_*xy*_ = 4.6%, the layer strain of the bottom layer relaxes again by nucleating an additional set of Moiré islands at the edges. The emergence of new islands pushes the old Moiré islands towards the center and also reduces the dimensions/periodicities of the islands. The correlation between the formation of Moiré islands and the reduction in layer strain is reconfirmed. The maximal displacement of the new Moiré islands is 0.41 Å, which is almost the same as those nucleated at an applied strain of *ε*_*xy*_ = 2.6%. The downwards displacement shows a maximal amplitude of −0.053 Å. With the emergence of additional islands, the repeated distance is reduced to 7.65 nm, which is consistent with the theoretical value of 7.69 nm. The edges of top layer are continued to push upward, and a set of Moiré islands are observed on the top layer as well. Thus, tensile strains imparted by the substrate on to a CVD-grown bilayer MoS_2_ terraced structure result in the formation of Moiré patterns as a strain relaxation mechanism through vertical displacements of the atoms.

**Table 1:** The maximal upward displacement, $\delta {z}_{\max }^{up}$, downward displacement, $\delta {z}_{\max }^{down}$, and periodicity of Moiré pattern, Δ_*r*e*p*_ during the tension.

Strains	Bottom Layer			Top Layer		
${{\boldsymbol{\varepsilon }}}_{{\boldsymbol{xy}}}$	${\boldsymbol{\delta }}{{\boldsymbol{z}}}_{{\bf{\max }}}^{{\boldsymbol{up}}}$ **(Å)**	${\boldsymbol{\delta }}{{\boldsymbol{z}}}_{{\bf{\max }}}^{{\boldsymbol{down}}}$ **(Å)**	**Δ**_re*p*_ (nm)	${\boldsymbol{\delta }}{{\boldsymbol{z}}}_{{\bf{\max }}}^{{\boldsymbol{up}}}$ **(Å)**	${\boldsymbol{\delta }}{{\boldsymbol{z}}}_{{\bf{\max }}}^{{\boldsymbol{down}}}$ **(Å)**	**Δ**_re*p*_ (nm)
1.5%	0.28	−0.032	N/A	0.087	−0.053	N/A
2.5%	0.39	−0.024	N/A	0.064	−0.072	N/A
2.6%	0.42	−0.036	13.09 (12.72)	0.13	−0.037	N/A
4.6%	0.41	−0.058	7.65 (7.69)	0.31	−0.034	N/A

The theoretical periodicity is given in the parenthesis. The displacements are in a unit of Å and periodicity is in a unit of nm.

**Figure 5:**
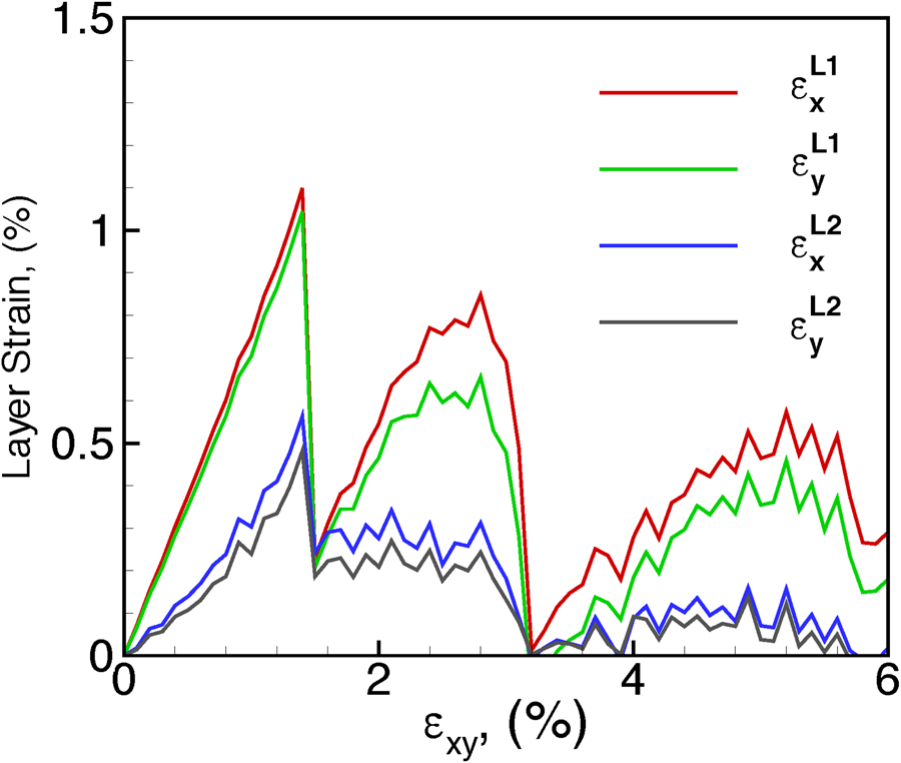
Local layer strain ${\varepsilon }_{x}^{bottom}$, ${\varepsilon }_{y}^{bottom}$, ${\varepsilon }_{x}^{top}$, ${\varepsilon }_{y}^{top}\,$as a function of applied strain *ε*_*xy*_ show the emergence and nucleation of Moiré island under biaxial compressive strain.

**Figure 6:**
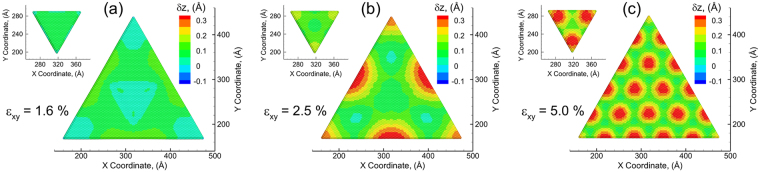
The vertical displacement map shows the nucleation of displacement islands and the formation of Moiré pattern in the top layer and bottom layer at different applied biaxial compressive strain: (**a**) 1.6%, (**b**) 2.5%, and (**c**) 5.0%.

However, it is not clear if such patterns are also observed under biaxial compressive strains. The biaxial compressive strain is therefore also applied to the triangular MoS_2_ bilayer by the substrate to understand the atomic scale strain relaxation response. The variation of layer strains (${\varepsilon }_{x}^{{L}_{i}}$, ${\varepsilon }_{y}^{{L}_{i}}$) for each individual layer (bottom and top) in the bilayer structure as a function of applied biaxial compressive strain (*ε*_*xy*_) is shown in Fig. 5, and the vertical displacement maps of bottom layer and top layer at different applied strain are presented in Fig. 6. Similar to the case of tension, the layer strains in the bottom layer and top layer present simultaneous fluctuation. The layer strains undergo sharp drop at an applied strain of *ε*_*xy*_ = 1.6%. Interestingly, as shown in Fig. 6(a), Moiré patterns are not observed at this strain. Instead, extensive upwards with a maximum of 0.14 Å (Table 2) are formed at the edges/sides of the bottom layer, while relatively small upward displacements (~0.02 Å) are observed at the corner and the center of the bottom layer. As a result, in the case of compression, the first decrease of the layer strain is contributed to extensive upwards displacement at the edges other than the nucleation of Moiré islands. After the first drop, the layer strains increase again with the increasing applied biaxial strain (Fig. 5). The layer strains in both bottom layer and top layer start to display significant anisotropic characteristic at this stage. It can be clearly seen that the layer strains in *x* direction are relatively larger than those in *y* direction in both layers. Such an anisotropy is not noticeably observed when applying tensile strains (Fig. 2). At an applied strain of *ε*_*xy*_ = 2.5%, the layer strains decrease again with the increasing applied strain, which is caused by the formation of Moiré islands as shown in Fig. 6(b). The Moiré patterns are first nucleated at the sides of the triangle. This is distinct from the case of tension, where the Moiré patterns are initiated at the corners (Fig. 3(c)). Unlike tension, there is no downwards displacement in both layers in the case of compression. The Moiré islands in the bottom layer are distinct from the atoms in adjacent region by presenting larger upwards displacement. The maximal upwards displacements (red regions) of these Moiré islands in the bottom layer are found to be 0.35 Å, while the minimal displacement in the adjacent region (green regions) is 0.038 Å. The periodicity of Moiré patterns is 17.92 nm, which is slightly larger than that of the theoretical prediction (16.83 nm). Meanwhile, the corners of top layer show upwards displacement with a maximal magnitude of 0.19 Å and minimum of 0.087 Å. Additional set of Moiré islands are observed to form at the applied strain of *ε*_*xy*_ = 5% (Fig. 6(c)), accompanied by the decrease in the layer strains (Fig. 5). As shown in Fig. 6(c), Moiré patterns start to form in the top layer as well at the applied strain of *ε*_*xy*_ = 5%, with a periodicity of 6.21 nm that is similar to the bottom layer (6.82 nm). The maximal displacement in the bottom layer at the applied strain of *ε*_*xy*_ = 5% stays the same (0.35 Å) as compared to that at the applied strain of *ε*_*xy*_ = 2.5%, while the minimal displacement increases from 0.038 Å (at the applied strain of *ε*_*xy*_ = 2.5%) to 0.074 Å (at the applied strain of *ε*_*xy*_ = 5%). As a result, with the increasing applied strain, the difference between maximum and minimal displacements become insignificant and the Moiré patterns become less distinguishable.

**Table 2:** The maximal upward displacement, $\delta {z}_{\max }^{up}$, minimal upward displacement, $\delta {z}_{m{\rm{in}}}^{up}$, and periodicity of Moiré pattern, Δ_re*p*_ during the compression.

Strains	Bottom Layer			Top Layer		
*ε*_*xy*_	${\boldsymbol{\delta }}{{\boldsymbol{z}}}_{{\bf{\max }}}^{{\boldsymbol{up}}}$ **(Å)**	${\boldsymbol{\delta }}{{\boldsymbol{z}}}_{{\bf{\min }}}^{{\boldsymbol{up}}}$ **(Å)**	**Δ**_re_ (nm)	${\boldsymbol{\delta }}{{\boldsymbol{z}}}_{{\bf{\max }}}^{{\boldsymbol{up}}}$ **(Å)**	${\boldsymbol{\delta }}{{\boldsymbol{z}}}_{{\bf{\min }}}^{{\boldsymbol{up}}}$ **(Å)**	**Δ**_re*p*_ (nm)
1.6%	0.14	0.02	N/A	0.072	0.048	N/A
2.5%	0.35	0.038	17.92 (16.83)	0.19	0.087	N/A
5%	0.35	0.074	6.82 (6.53)	0.34	0.12	6.21 (6.53)

The theoretical periodicity is given in the parenthesis. The displacements are in a unit of Å and periodicity is in a unit of nm.

Thus, Moiré patterns emerge in these stacked MoS_2_ few layered systems through upward/downward displacements to accommodate the elastic strains (tensile or compressive). The displacements are observed to originate at either the edges or the corners of the structures. Additional simulations with larger dimensions of these layers did not indicate any substantial changes in the emergence of Moiré patterns. These results suggest that MoS2 few layered CVD-grown structures when supported on a substrate, the mismatch strains between the substrate and the layers are likely to result in the formation of Moiré patterns.

## Conclusions

Molecular dynamics simulations are carried out to investigate the strain response of CVD-grown MoS_2_ structures at the atomic scales. The interatomic interactions between Mo/S atoms are optimized in the form of hybrid REBO/LJ potential to best capture the structural and energetic properties, as well as the strain configuration at the presence of edges. A bilayer MoS_2_ triangles over a MoS_2_ substrate layer is created to model CVD-grown structure with realistic edge configurations. The strain response at an applied tensile strain of 1.5% shows a “three-pointed star” shape in displacement map, which is astonishingly similar to the PL intensity map of the bilayer MoS_2_ triangle found in recent experiments. Besides, the MD simulations suggest that the strain subjected to the substrate is not able to completely transferred to the bilayered MoS_2_ sample, and the difference in the layer strain causes lattice mismatch, which results in the formation of Moiré pattern. The emergence of Moiré islands is correlated to the reduction in the layer strain. A new group of Moiré islands are nucleated every 2% applied strain in tension and 2.5% in compression, which results in a periodic reduction in the layer strain.

## Methods

### Molecular Dynamics

Large-scale MD simulations of model CVD-grown systems are carried out using LAMMPS^48^ with the interatomic interactions defined using the reactive empirical bond-order (REBO) potential^49^ combined with a Lennard Jones (LJ) potential. The time step for all simulations are defined to be 1 fs. All systems created are first equilibrated at 0 K for 10 ps at constant temperature and zero pressure (NPT ensemble using the Nose-Hoover algorithm), then the equilibrated structure is used as a strain free configuration for the following deformation simulations.

### Interatomic Potentials

The energy for an atom in the REBO formulation is calculated as: write this similar to the ripples paper.

4 $${E}_{REBO}=\frac{1}{2}\sum _{i\ne j}{f}_{ij}^{C}({r}_{ij})[{V}^{R}({r}_{ij})-{b}_{ij}{V}^{A}({r}_{ij})]$$

where ${r}_{ij}$ is the distance between atoms *i* and *j*, ${f}_{ij}^{C}({r}_{ij})\,$is a cutoff function and smoothly switches REBO to LJ, and ${V}^{R}({r}_{ij})\,$and ${V}^{A}({r}_{ij})$ are repulsive and attractive terms, respectively.

5 $${V}^{R}({r}_{ij})=(1+\frac{{Q}_{ij}}{{r}_{ij}})A{e}^{-\alpha {r}_{ij}},\,{V}^{A}({r}_{ij})=B{e}^{-\beta {r}_{ij}}$$

The intralayer vdW interaction is described by the LJ potential:

6 $${E}_{LJ}=4{\varepsilon }_{ij}[{(\frac{{\sigma }_{ij}}{{r}_{ij}})}^{12}-{(\frac{{\sigma }_{ij}}{{r}_{ij}})}^{6}]$$

To model strain response of triangular-shaped MoS_2_ 2D structures, it is essential to the interatomic potential is able to capture the correct strain configuration of individual layers. As discussed before, these stacked triangles exhibit more realistic and sophisticated edge configuration. As a result, the original potential parameters are re-optimized to better capture the strain response at the presence of edges/steps. The details are given in Supplemental Information.

### Data availability statement

Any data that is requested by the Editors and Reviewers will be made available upon request.

## Electronic supplementary material

Supplementary Information
