# Supplementary material for: Origins of Moiré Patterns in CVD-grown MoS2 Bilayer Structures at the Atomic Scales
Source: Sci Rep. 2018 Jun 21;8:9439. doi: 10.1038/s41598-018-27582-z (PMC6013503; doi:10.1038/s41598-018-27582-z)
Supplement: Supplementary file 1 — Supplementary Information [file 41598_2018_27582_MOESM1_ESM.docx]

SUPPLEMENTAL Figures and figure captions

**Title: Origins of Moiré Patterns in CVD-grown MoS2 Bilayer Structures at the Atomic Scales**

**Authors:** Jin Wang^1^, Raju R. Namburu^2^, Madan Dubey^3^, and Avinash M. Dongare^1^,*

1 Department of Materials Science and Engineering and Institute of Materials Science, University of Connecticut, Storrs, Connecticut 06269, USA

2 Computational and Information Sciences Directorate, U.S. Army Research Laboratory, Aberdeen Proving Ground, Maryland 21005, USA

3 Sensors and Electron Devices Directorate, U.S. Army Research Laboratory, Adelphi, Maryland 20783, USA

*** Corresponding Author:**

Avinash M. Dongare

Materials Science and Engineering, University of Connecticut, Storrs, CT-06084

Email: [dongare@uconn.edu](mailto:dongare@uconn.edu)

**The Optimization of Interatomic Potential**

The strain relaxation of the elastic deformation depends not only on the in-plane covalent bonds, but also on the vertical van de Waals interaction between interlayer atoms. The lattice constants and the elastic constants are chosen as the structural and energetic indicators, respectively. First, the pair-terms of the covalent parameters, *A, B, α, β* in Eq. (2) are tuned to provide accurate lattice constants and cohesive energy. Then the well-depth, *ε*_ij_, and the equivalent distance, *σ*_ij_, in the LJ potential are adjusted to match the elastic constants and bulk modulus. The modified parameters are presented in Table SI. In Table SII, the value of structural and energetic indicators calculated from the parameterization of Liang [1], Stewart [2], and this work are listed and compared to DFT and available experimental results. Although the structural indicators are similar, the cohesive energy of MoS_2_ molecular is remarkably improved by 23.54 % (23.0 %) as compared to Liang’s (Stewart’s) parameters.

The final and essential criterion of the optimization of the potential in this work is to reproduce the DFT-predicted strain configuration of CV-grown bilayer MoS_2_ structure with free edges as used in previous studies [3]. The bilayer edge structure is shown in Figure S1. The system in the 2*H* stacking sequence is rectangular in the basal (XY) plane with the thickness of the two layers to be equal (Y direction), but relatively smaller along the X direction. Three-dimensional periodic boundary conditions are applied in the calculations so that the bottom MoS_2_ layer is actually infinitely large along X and Y directions, whereas the top layer has a finite width in the X direction and infinitely long along the Y direction. A vacuum space of 20 Å thick is used along the vertical to prevent unphysical interactions between adjacent images along this direction. The strain response is investigated under loading condition of uniaxial tensile strain (*ε_x_* ≠ 0, *ε_y_* = 0, *σ_x_* ≠ 0, and *σ_y_* ≠ 0,) where *ε_x_* and *ε_y_* (*σ_x_* and *σ_y_*) are the *average* strains (stresses) of the entire system along the X and Y directions. The local strain maps [39] under an applied uniaxial strain of *ε_x_*=4 % as computed using DFT and MD simulations with different sets of parameters are shown in Figure S2. The strain configuration of the bottom layer and top layer are distinct, which is indicated by the different scale for the two layers. The strain in the bottom layer is comparable to the applied strain (4 %), while the strain in the top layer are significantly reduced to ~0.5 % due to the presence of edges. All the three potentials are capable to predict a relatively accurate strain configuration of the bottom layer. However, Liang’s (Stewart’s) parameters overestimate (underestimate) the residual strain of the top layer. For example, for the Mo atom in the center of the top layer, DFT, Liang’s, Stewart’s, and our parameters predict the local strain at the value of 0.64 %, 0.83 %, 0.46 % and 0.57 %. As a result, our parametrization will be used in this work for the interatomic potential of Mo-S system.

**Table SI:** Optimized parameters for the Mo-S interatomic potential. The energy is in the unit of eV, and the length in Å.

| **Parameter** | **Mo-Mo** | **S-S** | **Mo-S** |
| --- | --- | --- | --- |
| ***A*** | 123.5155 | 859.9026 | 401.7058 |
| ***B*** | 494.3735 | 1049.054 | 947.8501 |
| ***α*** | 1.075007 | 1.10775 | 1.192679 |
| ***β*** | 1.161003 | 1.126736 | 1.269738 |
| ***σ*** | 4.6 | 3.13 | 3.625741 |
| ***ε*** | 0.000586 | 0.01876 | 0.003315 |

**Table SII:** The lattice constants and elastic properties of bulk MoS_2_ from DFT, experiments and MD calculation with different parameterization.

|  | $\boldsymbol{a}$ **(Å)** | $\boldsymbol{c}$ **(Å)** | $\boldsymbol{d}_{\boldsymbol{Mo}\mathbf{-}\boldsymbol{S}}$ **(Å)** | $\boldsymbol{d}_{\boldsymbol{S}\mathbf{-}\boldsymbol{S}}$ **(Å)** | $\boldsymbol{d}_{\boldsymbol{int}}$ **(Å)** | ***E*_coh_ (eV)** | **C_11_ (GPa)** | **C_33_ (Gpa)** | **B_0_ (GPa)** |
| --- | --- | --- | --- | --- | --- | --- | --- | --- | --- |
| **DFT** [3] | 3.16 | 12.11 | 2.4 | 3.12 | 2.94 | -16.01 | 218.8 | 47.3 | 37.8 |
| **Exp.** [4],[5] | 3.16 | 12.29 | 2.42 | 3.17 | 2.98 | N/A | 238 | 52 | 53.4 ^c^ |
| **Liang­** | 3.17 | 12.18 | 2.45 | 3.24 | 2.85 | -21.67 | 269 | 50 | 42.41 |
| **Stewart** | 3.17 | 12.17 | 2.44 | 3.24 | 2.85 | -21.58 | 255.32 | 36.62 | 32.16 |
| **this work** | 3.11 | 12.03 | 2.39 | 3.15 | 2.87 | -17.9 | 242.88 | 50.58 | 41.91 |


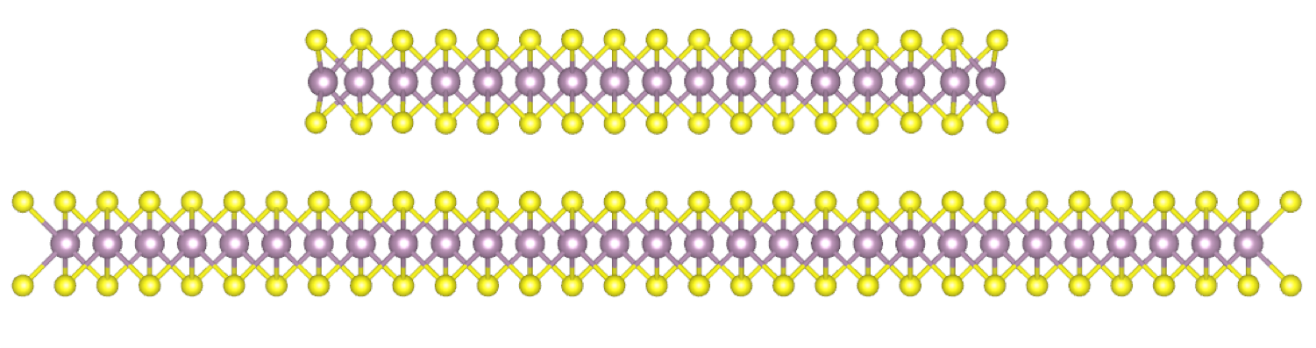


**Figure S1:** The front view of the as-grown MoS_2_ bilayer structure used in this study.


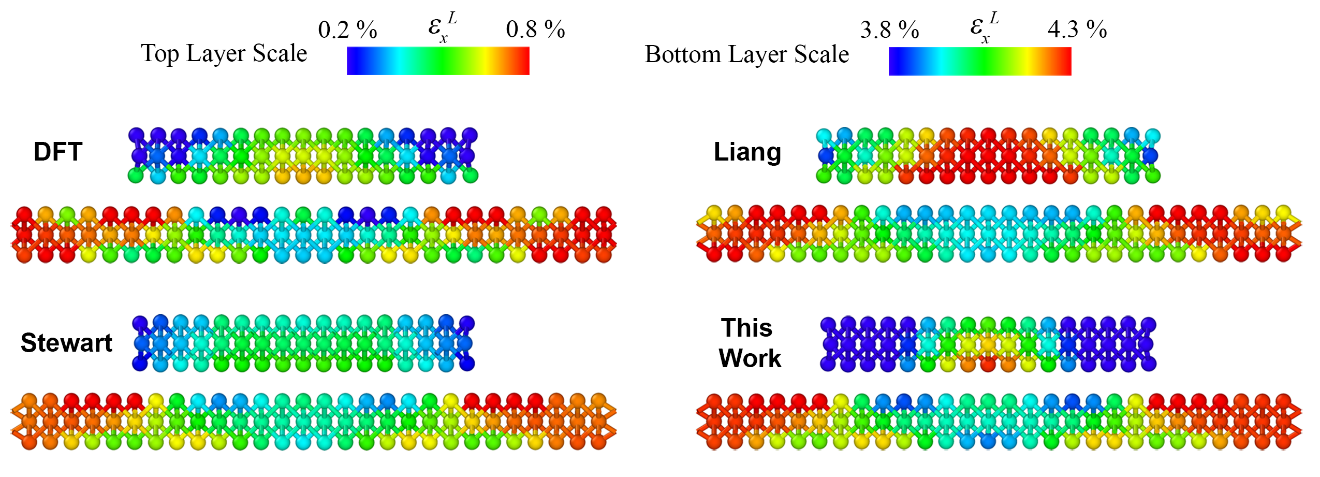


**Figure S2:** Comparison of Liang’s [1], Stewart’s [2], and our parameterization of Mo-S interatomic potential with DFT results: local strain ($\varepsilon_{x}^{L}$) map of a bilayer MoS_2_ system under an applied uniaxial strain $\varepsilon_{x}$=4 %.

**References**

1. T. Liang, S. Phillpot, S. Sinnott, Parametrization of a reactive many-body potential for Mo–S systems. *Physical Review B* **79**, (2009).

2. J. A. Stewart, D. E. Spearot, Atomistic simulations of nanoindentation on the basal plane of crystalline molybdenum disulfide (MoS_2_). *Modelling and Simulation in Materials Science and Engineering* **21**, 045003 (2013).

3. L. Dong *et al.*, Edge effects on band gap energy in bilayer 2H-MoS2 under uniaxial strain. *Journal of Applied Physics* **117**, 244303 (2015).

4. J. L. Feldman, Elastic constants of 2H-MoS_2_ and 2H-NbSe_2_ extracted from measured dispersion curves and linear compressibilities. *Journal of Physics and Chemistry of Solids* **37**, 1141-1144 (1976).

5. E. Selvi, Y. Ma, R. Aksoy, A. Ertas, A. White, High pressure X-ray diffraction study of tungsten disulfide. *Journal of Physics and Chemistry of Solids* **67**, 2183-2186 (2006).
